# A qualitative study to explore the attitudes of women and obstetricians towards caesarean delivery in rural Bangladesh

**DOI:** 10.1186/s12884-018-1993-9

**Published:** 2018-09-12

**Authors:** Tahmina Begum, Cathryn Ellis, Malabika Sarker, Jean-Francois Rostoker, Aminur Rahman, Iqbal Anwar, Laura Reichenbach

**Affiliations:** 1 Health Systems and Population Studies Division, International Centre for Diarrhoeal Disease Research Bangladesh (icddr,b), Dhaka, Bangladesh; 2 Department of Family Practice, University of British Columbia, Vancouver, BC Canada; 3 James P Grant School of Public Health, BRAC University, Dhaka, Bangladesh; 4 Institute of Public Health University, Heidelberg, Germany; 5 Population Council, Washington, DC USA

**Keywords:** Caesarean delivery, Qualitative study, Perception, Attitude, Decision-making, Rural Bangladesh

## Abstract

**Background:**

Caesarean section is a lifesaving surgical intervention for women and their newborns, though overutilization is a public health concern. The caesarean rate in Bangladesh is approximately 23% overall, and in private facilities it is over 70%. It is essential to know both the supply side (obstetricians) and demand side (parturient women) views on caesarean birth in order to formulate specific interventions to address the escalating rate of caesareans.

**Methods:**

This qualitative study took place in Matlab, a rural sub-district in Bangladesh. We interviewed women attending their 3rd antenatal visit, those with recent caesareans, and obstetricians from both public and private health facilities. In total there were twenty in-depth interviews and four focus group discussions. Study participants were asked about their preferences on birthing mode and knowledge of the caesarean section process. Thematic data analysis was done following a deductive approach.

**Results:**

Women from this rural community had a strong preference for normal vaginal birth. However, they were willing to accept the attending health care provider’s decision for caesarean birth. Antenatal care sessions did not provide information on the medical indications for caesarean section. Furthermore, some women had the misconception that episiotomy itself is a ‘small caesarean.’ Primary health care providers and clinic agents (brokers) had a strong influence on women’s decision to choose a health facility for giving birth. However, obstetricians, having a preference for caesarean section, were receiving more patients from these brokers which may be an important reason for the high rate of clinically non-indicated caesareans at private hospitals in Bangladesh. Improper labour monitoring and inadequate staffing at health facilities were additional influences on the preference for caesarean section. However, critical knowledge gaps were also observed among study obstetricians, particularly with regards to the indications for and timing of elective caesarean sections.

**Conclusion:**

There is a need to educate women about the advantages and disadvantages of different birthing modes to ensure their active participation in the decision making process. Strong policy regulations are needed to ensure legitimate decision making by obstetricians regarding mode of birthing.

**Electronic supplementary material:**

The online version of this article (10.1186/s12884-018-1993-9) contains supplementary material, which is available to authorized users.

## Background

Caesarean section is a life-saving surgical intervention for women and their newborns though its recent overutilization is a global public health concern [1, 2]. On average, there is a 4.4% annual rise in caesarean section rates worldwide, with Asian countries having the second highest annual increase during the period 1990–2014 [1]. In this context, the global recommendation of a 10–15% national caesarean rate has been critiqued and a revision urged [3]. A more recent multi-country survey conducted in 178 WHO member states has suggested that the population level caesarean section rate should not exceed 19%, as increased levels of neonatal and maternal mortality have been reported above this level [2]. Additionally, unnecessarily high caesarean rates have negative implications [4] at the individual, family, and national levels in terms of women’s well-being, health expenditure, and efficient use of resources [1].

When considering the reasons for rapidly increasing caesarean section rates, non-clinical factors have emerged as equally important factors as clinical factors [5]. Lower fees for vaginal birth, fear of litigation, and patient’s requests for the procedure are some of the non-clinical reasons for physicians to conduct caesarean sections [6]. Women who prefer caesarean birth consider vaginal birth to be a more painful and dangerous procedure, without considering the negative consequences of un-necessary surgical intervention [7]. Women with higher economic status and more formal education are more likely to make a self-request for caesarean section [8, 9]. Conversely, poor women with little education are presumed to have inadequate knowledge about caesarean section procedures [10] which is considered a significant barrier to involving women and their families in decision making related to the mode of birth [7]. As a result, service providers particularly the attending physicians are evolving as the solo decision makers regarding mode of birth, in low-income settings [6, 7].

However, physicians’ decisions also differ based on the location of clinical practice. Doctors working in a public sector facility assess caesarean indications based on clinical guidelines, while private practitioners are more concerned with litigation issues and have a tendency to perform more caesarean sections for making more business out of this procedure [11, 12]. This attitude might explain the increasing caesarean rate in for-profit private health facilities. For example, in Brazil the caesarean rate was 72% in the private sector, compared to 31% in the public sector [13].

In Bangladesh, maternal health has evolved within an extensive national health care delivery system that includes peripheral level health posts (community clinics) and health centres (union health sub-centres and health and family welfare centres) supported by primary, secondary, and tertiary level hospitals situated at sub-districts, districts, and regional levels respectively. Different cadres of technical and lay community health workers are working for maternal health that include physicians, nurse-midwives; family welfare visitors (FWV) and community skilled birth attendants (CSBAs). At the most peripheral level, the CSBAs and FWVs are the main maternal healthcare caregivers who provide services both from health-posts and health centres, and through home-visits. At the government hospitals level nurses and midwives are the main caregivers for vaginal deliveries. Physicians work mostly at sub-district, district, and medical college hospitals and are responsible for managing complicated vaginal and caesarean births [14]. Until recently, there was no accredited midwifery professionals in Bangladesh educated to the standards of the International Confederation of Midwives (ICM) and WHO [14]. During the Millennium Development Goal (MDG) era, midwifery education drew special policy attention in Bangladesh and a new strategic direction was initiated resulting in a three-year diploma in midwifery program launched in 2013 by the Ministry of Health and Family Welfare with technical support from UNFPA and WHO. Six-month post-basic midwifery training for existing public sector nurses was also organized as an interim effort to produce a critical mass of ICM/WHO standard midwifery professionals [15]. To date, 800 midwives have been accredited at a diploma level, and the vast majority of them have been deployed in district and sub-district hospitals [15].

In line with health related Sustainable Development Goals (SDG) targets, the government of Bangladesh is committed to improve access to both basic and comprehensive emergency care services to reduce Maternal Mortality Ratio (MMR). However, progress is slow; the Bangladesh Demographic Health Survey (BDHS) 2014 shows that the majority of births (63%) still take place at home, mostly with unskilled birth attendants [16]. Despite poor progress in achieving high skilled attendance rate, the caesarean section rate increased from 4% in 2004 to 23% in 2014, contributed mainly by the for-profit private health sector [16].

Experience from high-income settings suggest that exploring obstetricians’ and women’s attitudes towards caesarean section might be a productive way to guide policy in reducing un-necessary caesarean sections [17]. However, such information is missing in a number of developing countries such as Bangladesh. To bridge the knowledge gap in this area, the present study attempts to explore the attitudes of both women and obstetricians towards caesarean section birth in a rural area of Bangladesh.

## Methods

### Study design, setting and participants

We conducted this qualitative study in 2012 in the rural setting of Matlab, a sub-district of Chandpur district in Bangladesh. Matlab was selected as the study site because the proportion of births taking place in health facilities (80%), as well as caesarean section rate (29%) in this area was much higher than the national average of 37% facility delivery rate and the 23% caesarean section rate [18]. We used in-depth interviews (IDIs) and focus group discussions (FGDs) to investigate both client and provider views on caesarean section as a mode of birth in a low-income over-medicalization contexts.

The study participants were currently pregnant women and recent post-caesarean mothers representing the demand side, and obstetricians working in public and private health facilities representing the supply side. Study participants were recruited from six health facilities, three public and three private. Public health facilities were chosen in such a way that covers a range of referral facilities that included one district hospital, one sub-district level Upazila Health Complex, and one union level Health and Family Welfare Centre (UHFWC). From the private sector two ‘for-profit’ private hospitals and one ‘not-for-profit’ maternity clinic were selected as study sites. All private facilities were situated in Maltab township area. Of the six study facilities, the public sector district hospital and two of the ‘for profit’ private facilities had capacity to perform caesarean sections while the remaining four were basic emergency obstetric care facilities where only vaginal births took place.

### Data collection

#### Women’s participation

To explore women’s perspectives about caesarean section four focus group discussions (FGDs) and 14 in-depth interviews (IDIs) were conducted. FGDs were conducted with pregnant women during their third trimester of pregnancy while attending an antenatal care (ANC) visit in the study hospitals (public or private). In total 26 pregnant women participated in four FGDs. The third stage of pregnancy was chosen because women usually develop a birth plan by this time, as their expected date of delivery (EDD) is approaching [19]. The FGD sessions were conducted in a separate room in the study hospitals after completion of their scheduled ANC consultation.

A purposive sampling method was used to find the desired study participants from the targeted health facilities. Homogeneity of the participants in the focus groups was maintained in terms of education, socio-economic status, and area of residence. However, diversity in terms of parity, obstetric experience, and other socio-demographic characteristics was maintained while selecting FGD participants.

We also conducted fourteen IDIs with post-caesarean women, nine from private clinics and five from public sector health facilities. In private sector hospitals, we organized IDIs with post-caesarean women on their 4th postoperative day which took place in their hospital bed. Since no post-caesarean mothers were available in the public sector hospitals during the data collection period, women who had a caesarean in the Chandpur District Hospital within the last 42 days of the data collection period were tracked and sampled for interviews. We conducted IDIs with five such post-caesarean women and the interviews took place in the local health sub-centers where they came for scheduled postnatal checkups.

#### Obstetricians’ participation

We conducted six IDIs with obstetricians, three in public facilities and three in private hospitals. Only currently practicing obstetricians with advanced post-graduate training in obstetrics and gynaecology (training for more than one year) from study hospitals were included. A convenience sampling method was used where obstetricians available in the study health facilities during data collection period were approached for an interview. The interview took place in the obstetrician’s own visiting room in the hospital after regular office hours.

Each interview was forty-five to sixty minutes in length. Data collection was continued until saturation was reached. The data collection team comprised of the principal investigator (PI) and two experienced research assistants (RAs). All interviews were conducted by the PI while the RAs took notes. Each RA alternated with the other for each interview and did the same for data transcription and development of analytic memos.

We developed interview guidelines first in English and then translated them into Bangla which was finalized after field-testing in a similar rural setting to confirm the content and to identify any missing themes. Three separate guidelines were used to direct the interview session with the three different type of study participants. The interview guidelines are added as supplementary file (Additional file 1) with this manuscript.

All IDIs and FGDs were tape-recorded, with verbal informed consent, except one with an obstetrician from a private clinic who did not permit the audio recording. In that case, detailed notes were taken during the interview.

During the interview with women’s, their perceptions and attitudes regarding caesareans, preferred mode of birth, source and level of knowledge, cultural beliefs, and factors influencing decision-making about mode of birthing were considered. When interviewing obstetricians, their medical knowledge on indications for caesarean section, pros and cons related to each mode of birthing, perceived non-medical reasons to choose caesareans by fellow colleagues, and fear of litigation, were explored.

#### Ethics approval and consent to participate

Ethical approval was obtained from the Ethical Review Committee of the James P Grant School of Public Health, BRAC University. Informed oral consent was obtained from all the participants and permission was obtained from hospital authorities for using the hospital premises for study data collection. The consent form that was used for taking permission from study participants is attached with the manuscript as Additional file 2. This consent form detailed out the interview procedure, risk and benefit of participating in the study in front of the interviewee. Ethical Review Committee of JPGSPH reviewed the consent form and gave permission for taking Verbal informed consent considering the rural culture and related constraints on taking written consent. However, the Relevance, Appropriateness, Transparency and Soundness review guidelines were used to check the completeness of all the required qualitative study activities while preparing this manuscript [20].

#### Data analysis

We undertook a thematic data analysis using a deductive approach developed by the National Centre for Social Research, Framework analysis [21]. Data collection, transcription, and analysis were undertaken on an iterative processes. Initial transcriptions were in Bengali which were then back translated to English before coding [22]. WHO recommended “onwards backwards technique” for qualitative data transcription in between two languages was followed [23]. A list of a-priori codes were developed based on research themes. (The definition of each code and sub-codes with the condition when to use and not to use is mentioned in the code book which is attached as Additional file 3). The study findings were arranged systematically in matrices using those A-priori codes, which helped to identify the recurrent themes between participants. The summarized findings were compared and contrasted under themes recorded in the matrices [24]. Women’s attitude towards caesarean section was compared with obstetricians’ views, and pregnant women’s views towards caesarean section was compared with post-caesarean women’s recent experiences. The same themes which were commonly being reflected across the participants were grouped together. However, divergent but relevant themes were also reported separately. Intra-coder reliability was reported as 80% when checked between one research assistant and the principal investigator’s initial coding of two interviews and one focus group. Disagreement over coding was handled through re-reading interview data and further discussion between researchers.

## Results

### Study participants’ background information

#### Women participating in FGDs (*N* = 26)

The average (mean) age of pregnant women who participated in the FGDs was 23 years. The education status ranged from below primary to completed higher secondary level and four of them were studying at the bachelor level. The majority of them were housewives. Regarding place and mode of last birth, ten births took place at home with TBAs, four were vaginal births at a health facility; and two were caesarean birth and the remaining ten were primigravidas.

#### Women participating in individual interviews (*N* = 14)

The ages of the 14 post-caesarean mothers ranged between 16 and 37 years. Of them, six had below higher secondary level, three had secondary level education, and five had no formal education. Two post-caesarean women in private hospitals received no ANC while the others received, on average, three ANC visits during their last pregnancies. All post-caesarean IDI participants were housewives. The average monthly family income of women having caesareans at a public hospital ranged from 77 USD to 192 USD, while those from private hospitals ranged from 128 USD to 384 USD. The direct cost for a caesarean ranged between 77 USD and 128 USD in public hospitals and between 282 USD and 320 USD in private clinics.

#### Obstetricians participating in individual interview (*N* = 6)

The work experience of obstetricians ranged from four and a half to twenty years. All public sector obstetricians interviewed were involved in private practice after office hours.

The summary of socio demographic characteristics of all three categories of study participants are mentioned in the Additional file 4.

The details of FGDs and *individual interviews* in terms of number of participants, and location of each event is presented in Table 1.

**Table 1:** Data collection methods and participants

Methodology	Participant type		Number of interview/discussion session	Number of participants
Focus Group Discussion	Women	Pregnant women from private facilities	2	6
		Pregnant mothers from public facilities	2	7
Total			4	26
In depth Interview	Women	Post-caesarean mothers from public facilities	5	
		Post-caesarean mothers from private facilities	5	
			4	
	Obstetricians	Public health facilities	2	
			1	
			1	
		Private health facilities	1	
			1	
Total			20	

### Study themes and findings

**Fig 1.**
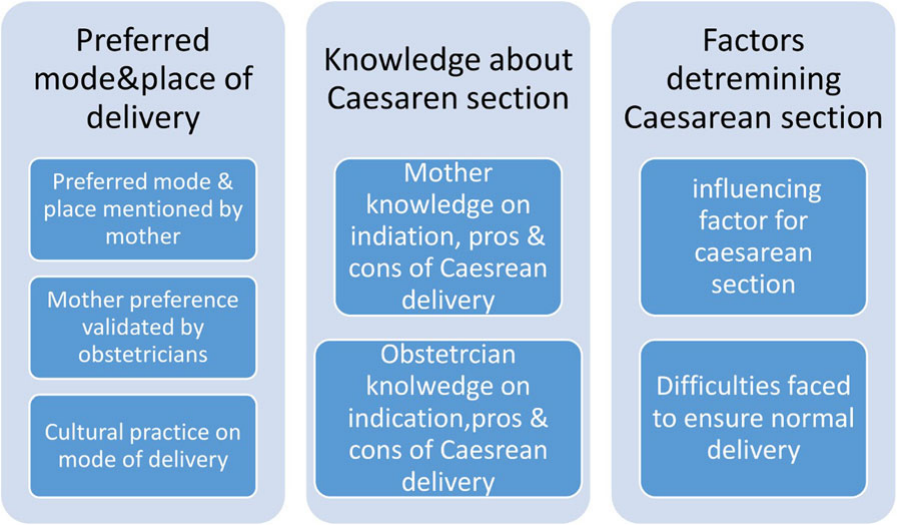
Study themes and sub-themes

Perceptions and attitudes regarding caesarean birth among pregnant and post-caesarean women were explored using six major codes: preferred mode of delivery, preferred place of delivery, knowledge about caesarean section, influencing factors, difficulties faced, and cultural practices. The obstetricians’ attitudes were investigated using three major codes: knowledge about caesareans, difficulties faced during vaginal birth, and women’s preferences. (The summary of study findings under all different codes are presented in Additional file 5) Subsequently, all the study findings gathered under these nine different codes were merged into three common themes: **preferred mode and place of birth**, **knowledge about caesarean, and factors determining decisions to perform caesarean section**. Under the theme of ‘preferred mode and place of birth*,’ the codes* women’s preference on place of birth, mode of birthing, and the reasons behind their attitudes were merged with obstetricians’ experiences of talking with women about their wish for normal delivery. The theme ‘knowledge about caesarean’ was used to group the knowledge of both women and obstetricians regarding the facts of caesarean births. Finally, the theme of *‘*factors determining decisions to perform caesarean sections*’* included the findings gathered under codes ‘influencing factors’ and ‘difficulties faced’ by the post-caesarean mothers and the obstetricians (Fig. 1)*.*

### Preferred mode and place of delivery

Both pregnant and post-caesarean women in this study expressed their preferences for vaginal birth at home where TBAs are the main delivery caregivers. Even if the cost of both normal vaginal and caesarean deliveries was assumed to be the same, they preferred a vaginal birth to a caesarean. The reasons included faster postpartum recovery and living in extended families where elderly members who were the main decision makers, had a strong preference for vaginal birth. Obstetricians also mentioned women’s preference, including those working outside of their homes, for vaginal birth in this community. When asked about attitudes and preferred mode of birth among working and educated women, obstetricians mentioned that home birth is preferred among these women as they still rely on elder women’s decisions in the family. A twenty-five year-old pregnant woman, during an FGD, commented on why having a caesarean is difficult and painful.

*“We need to carry paddy [rice] bundle over our head and need to feed our cattle. If suddenly rain comes we have to take the cut [rice] paddy from the yard to inside the house. In that case if I would have caesarean section, I will not be able to run. Women’s lives become really handicapped after having this operation” (Quote from pregnant woman)*

Perceived ‘health hazards to the women and her baby due to caesarean section’ were mentioned as another reason for preferring vaginal birth. Respondents expressed their views that caesarean babies are more prone to pneumonia and post-caesarean incisions remain painful for a long time. Obstetricians had similar views regarding incision pain; they mentioned that women who had caesarean sections visited their clinics with incision pain even six months post-surgery.

However, women in-general, were not rigid about their preferences but rather willing to accept physician’s decision for a caesarean, if deemed necessary. A twenty five-year old post-caesarean woman from a private health facility expressed her concern*,*

*“Before my case, none of my family members ever attended hospital for birthing purpose. As a part of the tradition, I also went to my natal home and they called our family ‘daima’ [traditional birth attendant]. When labour pain started, my baby defecated inside the uterus and instantly she referred me to this hospital. Consequently caesarean delivery saved my baby’s life.” (Post caesarean mother)*

Contrastingly, three of the twenty-six mothers participating in the FGDs showed a positive attitude toward caesarean section. One pregnant women mentioned:

*“I prefer Caesarean delivery. I heard baby delivered by caesarean section had a healthier brain compared to normally delivered baby as it does not stay in the birth canal for a long time”.(Quote from pregnant woman)*

One obstetrician from a public hospital mentioned that mothers demand caesarean sections for stillbirths. Considering it as unjustified on medical grounds, she commented:

“*We never recommend caesarean section for fresh stillbirth. Rather we try to induce normal labour through medication. However, women often consider the dead fetus as a poison within the womb and request for caesarean section within the shortest possible time. I get surprised having similar request from multiple cases in the recent past where my counselling did not work” (Quote from Obstetrician)*

With regards to a health facility preference (public or a private)., the majority of the pregnant and post-caesarean women had no specific preference. Rather they relied completely on the attending health care provider’s to choose the health facility.

### Knowledge on caesarean section

Women had limited knowledge and several misconceptions about caesarean section. They perceived there to be two types of caesarean section: one, ‘*small caesarean’* where women deliver normally (vaginally) with a cut in the perineum (episiotomy), and the second, ‘*large caesarean’* where the abdomen is cut under anesthesia to extract the baby.

One multiparous, post-caesarean mother from a private hospital said:

*“I am so happy that I have ‘large caesarean’ section this time. I had to suffer a lot … such as pain & itching in my private parts … even I had pain during sexual intercourse for a long time, since my last baby was delivered vaginally with ‘small caesarean’ section.” (Quote from Post caesarean mother)*

However, none of the pregnant women received any information about the medical indications for caesarean section, nor its benefits and risks, although they were attending the third trimester antenatal checkup on the date of the FGD. They received information about caesarean section mostly from relatives and neighbors who had experienced a caesarean section in the past. They thought caesarean birth was becoming a common event as more and more private clinics and hospitals were being established in their neighborhood.

*“The word caesarean section arose from the hospital. As, now-a-days number of hospitals has increased, caesarean birth has also increased. More mother and fetus used to die earlier but currently caesarean birth is saving lives of both, though sometime doctors do caesarean section unnecessarily for their financial interest.” … (Quote from Pregnant woman)*

When the pregnant women were asked to list the common clinical conditions for which their friends or relatives had undergone a caesarean, the top three causes were previous caesarean, less fluid, and baby defecating in the uterus.

On the other hand, obstetricians stated that they chose to not overburden the mothers with caesarean related information unless the current pregnancy had clear indications to deliver the baby surgically. All of them mentioned that although risks exist with operative procedures such as caesareans, it decreases maternal and neonatal deaths. Two (out of six) obstetricians in the study mentioned that having a vaginal birth attended by an inexperienced health care provider even in a hospital setting carries more health hazards than having a caesarean section.

When asked about common clinical reasons for performing caesareans, obstetricians mentioned two types: emergency and planned. Indications for emergency caesareans mentioned by all six obstetricians were similar. However, their responses for the cause of planned caesareans varied. While half of them mentioned that caesareans performed based on patient request are called ‘elective’, other obstetricians said that certain clinical conditions require a caesarean to be performed electively. However, while validating the obstetrician knowledge against the National Institute of Clinical Excellence (NICE) guideline recommended by the international expert committee, certain knowledge gap has observed. After excluding Cephalo Pelvic Disproportion (CPD), all the indications mentioned by obstetricians as planned C-section were in the contraindication list of NICE. The complete list of indications of planned Caesarean section mentioned by the study obstetricians are presented in the right column of Table 2 and the recommendation made by NICE on similar clinical conditions are presented in the left column of this table. For example, obstetrician believe women having breech pregnancy should be a case of caesarean delivery where birth order should not be a concern. On the other hand NICE guideline does not recommend planned caesarean section for breech pregnancy in higher order birth and for the first time mother without attempting to restore the fetal position through external cephalic version.

**Table 2:** Validation of obstetrician responses in contrast to National Institute of Clinical Excellence (NICE) guidelines on planned caesareans

Indication of planned caesarean section mentioned by obstetricians	Contraindication of planned caesarean section mentioned in NICE guidelines
• Previous caesarean • Breech presentation irrespective of parity • Twin pregnancy irrespective of parity and presentation • Preterm birth/ Small for gestational age • Cephalopelvic Disproportion (CPD) • Short stature of mother • Obese mother • Elective caesarean: to avoid conflict ✓ Hospital staff patients and well off family clients ✓ Patient with gestational diabetes ✓ Patient having bad obstetric history ✓ Primary/ secondary infertility ✓ Fear/can’t tolerate labour pain • Pre-eclampsia • Postdates pregnancy	• Previous caesarean • Primigravida breech without attempt of external cephalic version and breech in multiparous women • Twin pregnancy in cephalic presentation • Preterm birth/ Small for gestational age • Suspected CPD (that means short stature women could not be an indication) • Obese women even with BMI ≥ 50 • Elective caesarean (without obstetrical or medical indication) [36]

There were also mixed findings regarding the timing of elective caesareans. With the exception of one respondent, all of them stated that an elective caesarean could be done any time after 37 gestational weeks. One obstetrician said that he would only perform an elective caesarean two to three days before the expected date of delivery. The reluctance of obstetricians to choose the time for elective caesarean was commented on by a post caesarean mother who had an elective caesarean fifteen days before the expected date of delivery. Although she had no risk factors for vaginal birth, her obstetrician met her directly at the operation theatre once she was admitted to hospital.

“*I guess out of 100, only 10 lucky women could deliver normally. I wanted to deliver normally this time after having one ‘Caesar’ (caesarean section) five years back, but none of my doctors were willing to take the risk. Since there was no option other than caesarean, I came in this hospital 15 days prior to my given date of delivery and being delivered through C-section.” – (Quote from post caesarean mother)*

### Factors determining caesarean section

Community health care providers, from both the public and informal private sectors, played a significant role in the decision-making process regarding the mode of birth in this study. Being the first line health care provider; Community skilled birth attendants, Family welfare visitors, Traditional Birth Attendants and village doctors were the primary contact point for the pregnant women and their families at the community level. Almost all of the pregnant and post- caesarean mothers in the study mentioned visiting at least one of these community health providers before coming to the obstetrician.

The preference for a community health provider is shown in the following quote:

*“I will contact with ‘Apa’ at the time of delivery, she will try her level best to deliver my baby here. If my luck would not be good enough to deliver my baby at her hand, she will refer me to appropriate health facility. I usually go to her to get the address of doctor when any of my family members or me fall sick” (Quote from pregnant woman)*

A similar comment was made by several pregnant women in different FGDs. However, obstetricians in the study expressed a different view about community health providers. They claimed these community healthcare providers were serving as brokers for local private clinics and receiving commissions from these clinics for referring obstetric patients. However, the referral fees given to them varied according to the procedure, get higher commission for caesarean birth. So that they refer patients to the obstetrician who does more number of caesarean sections. Two of the study obstetricians claimed that some doctors perform caesareans without valid indications to upsurge their practice.

*“Here in this rural area patients are not choosing doctors based on academic degrees or experience, rather they choose doctors based on the recommendation from the referee (community health providers). Referrals were frequently for doctors who performed more caesareans instead of vaginal deliveries. I have heard that sometimes the tariff that they got is more than my operation charge.”- (Quote from Obstetrician)*

Obstetricians claimed that private clinics were using their agents, known locally as *‘dalals’* (brokers) to take the birthing women away from public hospitals. One study obstetrician mentioned that sometimes the service providers in the primary health facility refer birthing women to the higher facility without any valid medical indication and often with misleading clinical findings indicating the need for caesarean section.

“*Once I received a referred patient with complaints of fetal distress and meconium for caesarean section, but on examination I found no evidence of such things. In contrast, the woman presented to me had progressive normal labour with normal fetal heart rate, maternal pulse and blood pressure was ok, and so she could be allowed to deliver normally. I allowed the woman to delivery by normal vaginal methods and that occurred successfully. But other doctors may not follow the same procedures, because most of the doctors meet patients in the operation theatre in the private clinic.” (Quote from obstetrician)*

Poor quality of labour monitoring was also a concern for the private clinics. An obstetrician from a private clinic stated that:

*“We usually do not take risks when there is 50/50 chance of vaginal delivery even in the private clinics, as nurses working there are reluctant to do regular follow up and are not competent to manage normal delivery. My whole career will be ruined for a single fetal death.” (Quote from obstetrician)*

Similarly, obstetricians working in public hospitals claimed that inadequate numbers of skilled human resources was the main constraint for provision of high quality maternity care services. Although the health facility was well-equipped logistically, caesarean section services were not available after office hours (2 p.m.) due to unavailability of obstetricians and/or anaesthetists. Moreover, only one duty doctor was available after office hours to manage emergencies in both 50-bed UHC and in 250-bed district hospitals, and the duty nurses were not competent to manage normal vaginal deliveries. Obstetricians mentioned that they perform caesarean section in public hospitals only during office hours.

A lack of respectful treatment towards women in labour was reported to be a factor influencing the choice of delivery mode. One of the post-caesarean mothers complained about the attending nurse’s unsupportive behavior as a cause of her self-request for a caesarean birth. A nineteen year-old post-caesarean mother from a private clinic expressed her frustration:

*“I was admitted with labor pain in this hospital at 11 a.m. The nurse examined me there and said that it was not labor pain but I got sweating with that extreme pain. She did per vaginal examination several times, it was also much embarrassing and painful. I was losing my patience but they did not speak a single pleasant word to me. Then I left the hospital by giving ‘risk bond’ and went for caesarean section in a nearby clinic at 7 p.m.” (Quote from post- caesarean mother)*

## Discussion

This study identified three key factors affecting the mode of birth: client’s attitude, obstetrician’s attitude, and health facility influence. Client’s attitude on Caesarean delivery was mostly influenced by the health care provider and the attending health facility type. Whereas obstetrician’s attitude does not depend on their medical knowledge rather more subjective to financial interest of the clinics or brokers from whom they receive patients. In some instances Obstetricians knowledge on caesarean indications did not match with international guidelines and recommendations. On the other hand, public health facilities had certain challenges to provide 24/7 emergency obstetric services which creates more dependency to private health sector. However, private health facility’s undue interest to do medically non- indicated caesarean section was also being critiqued by the study obstetricians.

In general, women in this rural community had strong preferences for normal vaginal birth. Financial access does not equate with autonomy around decision making for mode of birth among women who work for wages. They still rely on elderly women in the family as influential decision-makers. This custom has also been documented in a study about the reasons for using a TBA in Bangladesh [25]. Despite the strong cultural preference for vaginal birth, women did not demonstrate a negative attitudes towards caesareans; rather they perceived these surgeries as necessary to save lives. This attitude contradicts findings from an earlier qualitative study conducted in the same study area, which reported that women who had caesareans felt it was an insult to their identity as a mother [26]. Our study, on the contrary, suggests that non-supportive behavior of nurses in the health facility, fear of episiotomy during vaginal birth, and the risk of fetal death during labour were the main reasons for which participants made self-request for caesarean. Similar to this study, maternal request for caesarean delivery after having an episiotomy and subsequent experience of painful sexual intercourse in a previous pregnancy were mentioned by the women from Nigeria and Turkey [27, 28]. It has also been reported that obstetricians also believed that vaginal birth is associated with more perineal injury and sexual dysfunction [29]. Despite this, other research suggests that postpartum sexual functioning is not associated with the birthing mode [30]. In addition, episiotomy performed during vaginal birth as a means of protection against perineal injury has not been proven effective always. A recent study among a large cohort (22,800 births) confirmed that statistically significant amount of perineal injury occurred even with lower episiotomy rate (6.7% episiotomy rate) [31]**.** Given that episiotomy is not protective against perineal injury and in fact causes short and long term negative sequalae, birth attendants should refrain from this practice to avoid unnecessary fear of episiotomy among women willing to give birth vaginally.

Some women also mentioned the lack of kind, comforting, and respectful behavior towards them during labour. Since an individual’s pain tolerance varies, it is recommended that health care workers be compassionate and supportive towards women in labour to increase confidence in their ability to give birth vaginally [32–34]. In our study, women were not knowledgeable regarding the medical indications for caesarean section. The antenatal care provided in the third trimester did not include information regarding risks and benefits of caesarean and normal vaginal delivery modes of childbirth. Friends and neighborhood women who had similar experiences remained the major source of information. Women’s lack of health literacy about surgical birth was used by some peripheral level lay health workers and informal providers to convince women to have caesarean sections even in the absence of valid clinical indications. For example, meconium staining is not listed as an indication for caesarean in the NICE guidelines [35]. However, women in this study believed that caesareans done on the grounds of meconium stained liquor could save their baby and for that they were thankful to their physicians. This finding corresponds to one systematic review which determined that poor knowledge about caesarean section is the main reason for women requesting caesareans, and recommended the involvement of parturient women in informed decision making processes [34].

The obstetricians in the study were found to be more positive towards caesarean section compared to vaginal birth. Fear of litigation and their prior maternity experiences were of paramount importance in determining their attitudes. However, their knowledge of indications for caesarean delivery did not coincide with international recommendations as indicated by the recent NICE guideline [36] and the American College of Obstetricians and Gynecologists Guideline [37]. For example, other than primigravida breech and cephalopelvic disproportion, all other clinical indications provided as reasons for performing caesareans by the study obstetricians are not listed in either of these international guidelines [36, 37]. Additionally, the obstetricians were not certain of the recommended gestational age for planned caesarean sections. This study identified two women who were having an elective caesarean fifteen days prior to their expected date of delivery. In contrast, the NICE guideline has recommended the appropriate gestational age for elective caesarean section to be after 39 weeks. Elective caesareans performed at earlier gestational ages have been associated with adverse neonatal outcomes such as increased rate of infections, respiratory distress and more admissions to neonatal intensive care units [38]. In this study, physicians were reluctant to involve women in evidence-based informed decision making, and were unaware of internationally accepted guidelines. In this regard, some health experts mentioned that if the “ruling class” clinicians perform caesareans without valid clinical indications, this erroneous practice and corresponding attitude may be accepted as ‘normal’ among the general population [39]. Once a high caesarean section rate becomes ‘normal’ it is difficult to change provider attitudes and practices.

The attitudes of both women and obstetricians differ based on the type of facility - attending hospital, private, NGO, or public. Concerns about safety included lack of ability to monitor labour by hospital staff and inadequate staffing of the facility. Other than the NGO clinic, both the public and private hospitals’ mothers and obstetricians did not have confidence on the nurse competency to monitor normal labour and their ability to handle any birthing emergency. In Bangladesh, normal childbirth in public hospitals is mostly attended by nurses and there is not yet a functioning midwifery profession. Poor quality of childbirth care has been reported in public health facilities [11, 40]. To address this gap, Bangladesh is in the process of adding newly trained midwives to the maternal and neonatal health care team, who will hopefully be proficient in attending normal childbirth, thus allaying some of the concerns voiced by mothers and obstetricians [15].

This study also highlighted the perception of inadequate human resources to provide basic and comprehensive emergency obstetric care after office hours in public facilities. Inadequate staffing and logistics has been documented as a barrier in public health facilities in the context of other developing countries as well [6, 11, 41]. To compensate for this constraint in resources, a defensive obstetric practice has been observed among the study obstetricians, who are performing elective caesareans during office hours. However, evidence suggests that a caesarean done without a valid medical indication increases the chance of maternal death 2.84 times in comparison to vaginal birth [1]. Women and obstetricians in this study appeared to be unaware of this evidence.

Financial motives and avoidance of possible lawsuits are documented as major influencing factors for caesarean births in private sector health facilities. Obstetricians interviewed from private clinics stated that they sometimes feel the need to perform unnecessary caesareans to meet the clinic’s financial demands. In doing so, the community health care providers and clinic referral agents, performed ‘patient counseling for caesareans’ on behalf of clinic authorities and obstetricians in the private sector. Our study findings corroborate other study findings from rural Bangladesh that have also confirmed the presence of a broker or ‘*Dalal*’ to move pregnant women from public health facilities to private facilities [11]. Our study findings depicts that women had to pay for caesarean section services in the public hospitals. This is a potential threat to the movement towards universal health coverage (UHC) as the poor may become poorer to meet maternity expenses; this is especially true in Bangladesh where the main mode of payment for health services is out-of-pocket [42].

### Limitations and methodological considerations

It was definitely challenging to investigate both women’s and obstetrician’s views in the same study. While doing so, the strengths of the study include getting similar responses from both the caregivers and care receivers in regards to preferred birthing mode and quality of care in public facilities. Assumptions that most women choose caesarean sections is challenged, and the pain and fear that women experience after episiotomy was identified as a key reason for requests for caesarean birth.

While we are confident in the findings of this study, it was not without its limitations. The study setting and sampling strategy raise methodological issues. The study was conducted in Matlab, an area in Bangladesh known for its long history of public health interventions and research studies as well as the presence of icddr,b, an international health research organization well known to the community. It is possible that the presence of icddr,b researchers may introduce some response bias. Furthermore, it is possible that the study findings do not accurately represent women’s and obstetrician’s view from other areas in Bangladesh. While the objective of the study was not to produce nationally representative results, we did provide adequate contextual description allowing readers to determine whether the findings are relevant in other areas of Bangladesh and elsewhere in low and middle income countries [22].

## Conclusion

The preference of women for vaginal birth in this study was not the major factor determining their attitude towards caesarean section birth. Women had inadequate information about caesarean birth and were mostly dependent on their provider’s decision regarding mode of birth. The fear of episiotomy was strong among the study participants. The context and underlying reasons for this attitude and the methods and indications for performing episiotomies, require further investigation. The need for respectful behavior among health care providers, ethical clinical practice and education of women and their active participation in the decision making process have been highlighted in this study. Ensuring availability of 24-h obstetric services in public facilities and educating pregnant women about choices in the mode of childbirth, including indications for, and complications resulting from caesarean section, is a productive way to avert third party influence in promoting unnecessary surgical birth.

## Additional files

Additional file 1: Code book. Description of A-priori codes and sub-codes. This file detail out the A-priori code and sub code-list that includes definition, when to use and when not use for each of the codes and sub codes. (PDF 773 kb)

Additional file 2: Consent form. Consent form in English. The consent form details out the interview procedure, risk and benefit of participating in the study. (PDF 306 kb)

Additional file 3: Interview guidelines. Guidelines to conduct In-depth interview and Focus Group Discussion. Three separate guidelines were used for in-depth interview with post caesarean women, obstetricians and Focus group discussions with pregnant women. (PDF 419 kb)

Additional file 4: Demography data. Demographic details of study participants. This excel sheet detail out the socio- demographic characteristics of all study participants including age in range, sex, education, religion, occupation, family income, cost of caesarean service etc.. (XLSX 20 kb)

Additional file 5: Data display. Summary of study findings. The study findings are grouped under different color codes. (XLSX 26 kb)

## Abbreviations

Caesarean section: Caesarean
FWV: Family Welfare Visitor
icddr,b: International Centre for Diarrhoeal Disease Research Bangladesh
JPGSPH: James P Grant School of Public Health
NGO: Non-Governmental Organization
NICE: National Institute for Health and Care Excellence
SBA: Skilled Birth Attendant
TBA: Traditional Birth Attendant
UHFWC: Union Health & Family Welfare Centre
