# Supplementary material for: A qualitative study to explore the attitudes of women and obstetricians towards caesarean delivery in rural Bangladesh
Source: BMC Pregnancy Childbirth. 2018 Sep 12;18:368. doi: 10.1186/s12884-018-1993-9 (PMC6134512; doi:10.1186/s12884-018-1993-9)
Supplement: Supplementary file 1 — Code book. Description of A-priori codes and sub-codes. This file detail out the A-priori code and sub code-list that includes definition, when to use and when not use for each of the codes and sub codes. (PDF 773 kb) [file 12884_2018_1993_MOESM1_ESM.pdf]

Interview guideline for IDI & FGD

Socio demography of study women

IDI number:

Date:

Site/Place:

Time: Starts: _____:_____ End:_____:__________

SN	QUESTIONS	RESPONSE	NOTES/REMARKS
1	Name		
2	Age		
3	Religion		
4	Education		
5	Residence(village name)		
6	Occupation		
7	Husband occupation		
8	Husband education level		
9	House hold head		
10	Family Income	Self:<br>Household:	
11	How many antenatal check up taken		
12	From whom ANC taken		
13	parity		
14	Previous delivery history (mode of delivery)		
15	Cost for C-section (formal & informal)		
16	Hospital where recent C-section done		

RQ: 2 To document the attitude and experience related to C-Section among the women in post- operative period both in public& private hospital of rural Matlab.

- Was the decision to have a C/S yours or someone else?(Probe for doctors or any 3rd part interference)
- How do you feel after having C/S?(it will cover both physical economic,social view,sexual practice)
- What are the obstacles you face to come here?
- What was your plan about delivery?(mode,savings,site if it is c/s)
- why the doctors or mid wives prescribed you for c/s?
- What did you know about c/s before coming here?
- What type of arguments you had with the attending physicians and your family member on taking final decision for this c/s?
- From this surgery who benefitted mostly you or your baby? Why?
- After recovery have there been any change on your perception about c/s?
- Will you advise this surgery for your near one?
- When and why you will do so?
- If you will be pregnant in future, what mode you choose for you to deliver? why?

RQ1: To understand the perception regarding Cesarean section among pregnant women attend in the public and private hospital in Matlab.

- What do you know about c/s?
- Where can a women get this service in your area?(probe about having any fear on facility related to c/s,preferred site for c/s)
- When a women need to do C/S? (probe about doctors decision either in favour or disfavor of it?)
- Why they need to do it?
- From where you get to know about it?(ask about from ANC visit)
- do you know someone among your neighborhood or relatives, , who need to went for C/S?
- Why they are advised to do so?
- How those women and her family accept that decision?
- What may be the experience of women who have had CS? (physical.family tasks, risk on sexual practice in relation to)
- How are they treated by their family and community after the CS?
  -acceptance by the family and community
  -make her guilty
  -certain behavior during pregnancy is considered to have negative effect,perceived by the family members.

- In your understanding, what are the advantages of CS?
- In your understanding, what are the disadvantages of CS?
- If you will be advised to do so in your delivery what will you do?
- Why you will do so? Probe is there anyone other than her selves who play potential influence on decision making)
- With same cost which mode of delivery you choose?
- Do you know someone who has done c/s just for her convenience without any indication?
- If yes, why women are doing so?

Socio demography of doctor participants

IDI number:
Date:
Site/Place:
Time: Starts: ____:___________ End: ______:______

SN	**Questions**	**Response**	**Remarks**
1	Age		
2	Sex		
3	Employment status		
4	Post graduation degree/training	Year	subj
5	Working experience		
6	Monthly salary		

RQ 3: To explore the knowledge and attitude on Cesarean section among doctors of public and private hospitals in that study area.

- In which cases you need to choose C/S as mode of delivery in your practice?
- Can you share with me what does you mean by elective c/s?
- What would be your response towards women request for c/s? (probe either he is also getting these type of response from his respondants)
- What are the advantages and disadvantages of c/s in compare to c/s?
- What is the overall perception of c/s among your clients in this area?
- What type of difficulties do you face to motivate women for c/s?
- What are some of the factors that influence your decision to do a C/S? probe
    - Type of hospital
    - Availability of resources both surgeon, anaesthetesits, nurses, instrument to monitor labour and so on
    - On call doctors
    - Media pressure (legal issue)
    - Client pressure(fear of litigation)
- If you consider the c/s rate now and 5years before is there any change in this area?
- if yes, what are the leading causes?
- Are you practicing both in public and private hospital?
- Is there any variation in c/s rate in public and private hospital? What may be possible causes in your experience?
