# Supplementary material for: A qualitative study to explore the attitudes of women and obstetricians towards caesarean delivery in rural Bangladesh
Source: BMC Pregnancy Childbirth. 2018 Sep 12;18:368. doi: 10.1186/s12884-018-1993-9 (PMC6134512; doi:10.1186/s12884-018-1993-9)
Supplement: Supplementary file 2 — Consent form. Consent form in English. The consent form details out the interview procedure, risk and benefit of participating in the study. (PDF 306 kb) [file 12884_2018_1993_MOESM2_ESM.pdf]

Informed verbal consent form for study participant (doctor and patient)

Hello (Assalamualaikum/Nomoshkar). My name is Tahmina Begum, a student from BRAC University in Dhaka. I want to learn about your perception about caesarean section and how you feel about it as a mode of delivery and what other circumstances lead you to take decision to do that.

I am approaching you because you are a pregnant women at such stage waiting to take final decision on mode of delivery or undergo this procedure right now (for patient of C/S),or as a obstetric doctor your main responsibility to motivate people to do c/s when necessary(for IDI of doctors) . The in-depth interview will take about 30-40 minutes and group discussion will take about 1 hour. There are no major risks involved in this study and your participation will be completely voluntary. We will use tape-recorder to record the information given by you. During the study, only with your permission, we would also like to be able to take relevant photographs of you and your environment.

Any information that I will obtain in connection with this study and that can be identified with you will remain confidential. You can give us your consent verbally to participate in this study. Although you decide to participate, you are free to withdraw your consent and to discontinue participation at any time without penalty.

If you have any questions, please ask me. If you have any additional questions later,I will be happy to answer them. You can call me directly on my mobile +8801819463947 You can also communicate with Prof. Malabika Sarker, Chair of Ethical Review Committee, James P Grant School of Public Health, BRAC University, level-5; 68, Shaheed Tajuddin Ahmed Sarani, Mohakhali, Dhaka-1212 for any query regarding this research

Do you agree to participate in this study?

Date:
