# Supplementary material for: A qualitative study to explore the attitudes of women and obstetricians towards caesarean delivery in rural Bangladesh
Source: BMC Pregnancy Childbirth. 2018 Sep 12;18:368. doi: 10.1186/s12884-018-1993-9 (PMC6134512; doi:10.1186/s12884-018-1993-9)
Supplement: Supplementary file 3 — Interview guidelines. Guidelines to conduct In-depth interview and Focus Group Discussion. Three separate guidelines were used for in-depth interview with post caesarean women, obstetricians and Focus group discussions with pregnant women. (PDF 419 kb) [file 12884_2018_1993_MOESM3_ESM.pdf]

Description of A-priori codes

Study title: A qualitative study to explore the attitudes of women and obstetricians towards caesarean delivery in rural Bangladesh

---

1. **Preferred mode of delivery (PreMo)-**

a. Brief Definition: which mode of delivery women choose for herselves.

b. Full Definition: irrespective of their condition during labour, in which mode they feel comfortable to deliver. it is very much subjective not influence by other people. different from perceived need which is sometimes influence by other people also.

c. When to Use: what mode they prefer usually, after having one c/s does it vary, with same cost which method prefer and what is the reason to choose it.

d. When not to use: if they say, prefer it as because her doctor or other person prescribe it for her.

Example: choose normal vaginal delivery as because it is occurring normally, no need of intervention.

2. **Knowledge on c/s among patient (know pt):**

a. Brief definition- It will cover their basic understanding about this procedure.

b. Full Definition-women knowledge on c/s in comparison to normal vaginal delivery will assess here. In addition the advantages and disadvantagesof c/s on maternal and neonatal health will be also asked.

c. sub code(when to use & not to use)

source of information (**So In**): it will tell about where does they come to know about it as a mode of delivery

Indication of C/S **(In C/S)-** it is not expected that they will say the medical indications correctly just to explore their perception. But it will not cover their attitudes, positive or negative on it.

Safety vs risk with C/S **(Saf vs Risk**) it will only include safety and risk associated with each procedure. Not include the additional benefit or loss.

For example: NVD cause injury in perineum. C/S has no pain sensation as done by anaesthesia

where to go for it (**site)** – it will not include their preferred site. only their level of knowledge on the availability of resources in their vicinity will cover.

Who benefitted (Benefit Who) – if they consider c/s is necessary for some instances then to whom it is mostly necessary either mother or fetus.

3Influncing Factors For Patients (Inf Pt) –

a. Brief definition: the factors which influence the decision of pregnant and postnatal women the necessity of c/s.

b. Full Definition: this will explain the possible factors which influence their decision either they are prescribed it or not. On the contrary the difficulties will only include the factors after taking decision or prescribing it by doctors in emergency situation

c.Sub Code:

Female Autonomy (Fem Auto)-either women education or occupational status have any role.

Cultural Preferences (Cul Pref)- explore the cultural preference of mode of delivery in this area and find out any relation of it on decision making

Third Party Influence (Third Inf) –either doctors,midwives or any person like dalal put significant role on it and among these categories who are the most significant in their context.

Other factors (Oth Inf)- some other factors mention by the study participants.

4. Preferred site for c/s (Pref site)-

a. Brief Definition: which facility they prefer to go for c/s

b. Full Definition: If they are prescribed to go for c/s which facility they will choose for it either public or private and what are the reasons behind it.

c. When to use: choose pubic one as it is cheap, private facility have good care etc.

d. When not to use: if they are forced to choose any facility due to some reason it will not include here. Due to unavailability of doctors at night in public hospital they have to go in private facility.

5**. Difficulties face by patient (diff pt**) :

a.Brief Definition: It will only include the difficulties mention by the patient who need to go for emergency c/s.

b.Full Definition: this is basically cover the **experience** of post natal patient while accessing the services . This is different from the impact of C/S, which will describe the difficulties or advantages after having C/S.

c.sub codes:

unprepared (un prep) : due to emergency nature or lack of information on ANC visit have to suffer. While waiting for normal delivery get less information when they may need to go for C/S, unavailability of support person at that time

Cost: having no save money or out of capacity to meet expenses will include here. But different from the impact on family which is mainly cover effect of borrowing money in family.

Accessibility: sufferings related to distanced C/S facility.

other difficulties mention by patient (Other Diff)

6. Difficulties face by doctors-(diff doc)

Brief Definition: difficulties face by doctors while motivating women.

When to use: patient and family member's negative attitude towards C/S and doctors, to get the right person during counseling, less educated people are less receiptive.

When not to use: This is different from the influential factors, which is for both in favours or disfavours of C/S. But difficulties only cover the factors work against decision making for C/S.

For example: during emergency C/S dispute arise among natal and in laws house, no wants take decision on c/s.

7.Impact on family(Imp Fam)-

Brief Definition: How this surgery affect their family's livelihood.

Full Definition : After having c/s does they need to develop any coping strategy to meet up the daily need of the family.

When To Use: Need to sale asset, borrow money which in turn put pressure on earning member to work hard

When Not to Use: Way of managing immediate cost which will cover by cost in difficulties theme.

8.**Impact on women(Imp women)-**

a.Brief Definition: Effect of C/S on women.

b. Full Definition: physical sufferings of women, which is differ from risk and benefit related to C/S. while the Risk vs benefit related with the procedure itself, suffering related with the effect of any perceived risk or benefit in her life. This theme also include the effect on breast feeding practice, child care, sexual practice and specially how they are treated by their family and community

c.When To Use: c/s needs to long day for recovery so, cant take care of new born properly,NVD may lead to perineal tear which will interfere with sexual life,they are accepted by the community as separate entity as they had less tolerance to bear labour pain.

d.When not to use: NVD cause perineal tear,C/S related with wound infections

9. **Influential factors for doctors-(Inf Doc)**

Brief Definition: what are the factors influence the doctors decision in favour or disfavor of c/s

Sub code

- Type of hospital (Typ Hos)-does the decision on c/s vary in between public and private hospital, if so why?
- List of Resources (List Resorse)-what are the potential resources have the influence to take decision on c/s either emergency or elective both surgeon, anaesthetesits, nurses, instrument to monitor labour (increase or decrease the rate).

  For example: having more staff and modern facility like USG or CTG machine to monitor labor can make them more safe on decision.

- On call doctors (On Dctrs)- Effect of absence of on call physician in the decision of c/s.

  For example-preprogrammed c/s while with emergency facility can wait for while.

- Media pressure (Med Press) –in favour or disfavor of excess c/s and the effect of their coverage on your reputation.

- Client pressure (Pt Press)-patient demand for c/s influence their decision. if not how they takle patient request

10.Knowledge of Doctors-(know doc)

Brief Definition: Indication of C/S,what are the possible cause to increase the C/S rate
.
Full Definition : It will cover their knowledge on indication of C/S, advantages or disadvantages related with it and what they know about elective C/S. what They are knowing about current C/S status in their context, if it is rising why it is occurring.

When to use: Name of conditions when C/S necessary, elective C/S are doing in certain medical conditions or patient request and at what time of pregnancy it need to do, why C/S are rising.

For example: elective C/S are doing before full term pregnancy, placenta praevia, history of previous C/S need to deliver by this procedure.
